# Supplementary material for: Patterns of Intron Gain and Loss in Fungi
Source: PLoS Biol. 2004 Nov 30;2(12):e422. doi: 10.1371/journal.pbio.0020422 (PMC532390; doi:10.1371/journal.pbio.0020422)
Supplement: Table S1 — Also available at http://genes.mit.edu/NielsenEtAl/. (4.3 MB ZIP). [file pbio.0020422.st001.zip › NielsenEtAl/html/1229.html]

AN3889.1.NCU01904.1.MG02914.1.FG08943.1


```
 CLUSTAL W (1.82) Multiple Sequence Alignments - Introns Inserted


Sequence 1: NCU01904.1	265 aa
Sequence 2: MG02914.1	260 aa
Sequence 3: FG08943.1	287 aa
Sequence 4: AN3889.1	394 aa
Alignment Length: 401 aa
Number Identitical Residues: 131 aa
Alignment Score (without introns) 6514


MG02914.1 	-----MAANFFSLKDHTALITGGTRGIGAAVAIGLAEAGADVLLVQ0------RDESSTT
NCU01904.1	MTSTTSVQNMFSLEGHTALITGATRGIGQAVAIALAEAGADILLVQ0------RDETSTT
FG08943.1 	-----MSASFFSLQGQTALVTGGTRGIGQAVAIGLAEAGSDIILVQ0------RDTTSQS
AN3889.1  	---MTDVASLFSLSGKTALVTGGTRGIGAAMAIALAEAGADIILIQ0LTCDAQRDTSNTT
          	    :   .:***..:***:**.***** *:**.*****:*::*:*  :..:.** :. :

MG02914.1 	TRDAILALGRKSQIYVADLSSPESVAALTPKVLADGHQVRILVNCGGIQRRHKCEVFPDS
NCU01904.1	TLQAIQSLGRHAQIYKADLSSPTDVSSLVPRILSDGHTIRILVNCAGIQRRHPSHLFPDS
FG08943.1 	TKESIEKLGRKAFIYTADLSSQESVAALVPKVLADGHEIRILINCAGIQRRHPCEVFPDS
AN3889.1  	TRDQITRLGRKATIHAAELSDRSAVKAIIPTLVSQGHNPTILLNCAGIQRRHPSERFPDE
          	* : *  ***:: *: *:**.   * :: * ::::**   **:**.****** .. ***.

MG02914.1 	DFNE~VMQVNLGAVFTLTRDVGAHMLSLEPHPVTG--RRGSVVNYASLLSFQGGLTVPAY
NCU01904.1	DFAE~VMQVNLNAVFTLCRDVGAHMLGLDPHPVTR--RKGSVINFASLLTFQGGLTVPAY
FG08943.1 	DFNE0VIQVNLNSVFTLCRDVGAHMLNLEPSPVTG--RRGSIINFGSLLTFQGGFTVPAY
AN3889.1  	DWDE~VLNVNLTSVFTLCREFGAYILSLPESSFTPNKKKGSIINVASLLSYQGGITVPAY
          	*: * *::*** :**** *:.**::*.*   ..* ..::**::* .***::***:*****

MG02914.1 	AASKGAVAQLTKSFANEWTSRGVTVNAIAPGYIATDMNEALLKDEERLASISARIPAGR~
NCU01904.1	AASKGAVGQLTKSFANEWTSKGVTVNAIAPGYIETEMNEALLKDEERLKSISARIPAGR0
FG08943.1 	AASKGAVGQMTKSFANEWTAKGITVNAIAPGYIETDMNEALLNNPERLASITSRIPAGN~
AN3889.1  	AASKGGVAQLTKALSNEWVSKGINVNAIAPGYIDTDMNVALINDANRNAGIMARIPAGR~
          	*****.*.*:**:::***.::*:.********* *:** **::: :*  .* :*****. 

MG02914.1 	WGDPEDFKGTTVYLASKASGY~VSG----HVLVVDGGWMG-~------------------
NCU01904.1	WGSPEDFKGTAVYLASKASGY~VSG----HVLVVDGGWMG-~------------------
FG08943.1 	WGSPDDFKGTSVYLASKASGY0SKYPHIKSTACANKSSFG-~------------------
AN3889.1  	WGRPEDFKGPVVFLASEASSY~VSG----ELVTVDGGWMGS2LETSFVALTEEVGELLVS
          	** *:****. *:***:**.*  .         .: . :*:  .::  : :.. ..   :

MG02914.1 	-----------------------R------------------------------------
NCU01904.1	-----------------------R------------------------------------
FG08943.1 	-----------------------QTRHAVAIR-----DYCDLVDKTADDMQH--------
AN3889.1  	QKPFVYRFYFHITIYPSLYTAFNRTSAALFLTTTLRQATCHPVDRNTHLTTSPDSTSCPM
          	...         :  .:  :: .::  :    ::  .  .   . .:     ..:::.. 

MG02914.1 	----------------------------------------------
NCU01904.1	----------------------------------------------
FG08943.1 	----------------------------------------------
AN3889.1  	GNPRSSLVTTRTGNETPIKGNLIPPSSGPVLLRTTPQQQQQRRTLR
          	... ::  :: :...:. ...  ..::..    ::......  :
```
